# Author Correction: Synthesize high-dimensional longitudinal electronic health records via hierarchical autoregressive language model

**DOI:** 10.1038/s41467-023-43203-4

**Published:** 2023-11-21

**Authors:** Brandon Theodorou, Cao Xiao, Jimeng Sun

**Affiliations:** 1 University of Illinois at Urbana-Champaign, 201 North Goodwin Avenue, Urbana, IL USA; 2 Medisyn Inc., Las Vegas, NV USA

**Keywords:** Translational research, Computer science, Computational science

Correction to: *Nature Communications* 10.1038/s41467-023-41093-0, published online 31 August 2023

The original version of this article contained errors in Table 3 and in equations 7 and 8.

In the first column of Table 3, the text ‘er’ appears before HALO-Coarse; this should only read HALO-Coarse.

The error equation 7 only appears in the PDF version, and not in the HTML version; it incorrectly reads ${{{{\bf{H}}}}}^{(m)}={{{\rm{transformer}}}}\_{{{\rm{block}}}}({{{{\bf{H}}}}}^{(m-1)})\,\forall 2.22144ptm\in [1,M]$ and it should read ${{{{\bf{H}}}}}^{(m)}={{{\rm{transformer}}}}\_{{{\rm{block}}}}({{{{\bf{H}}}}}^{(m-1)})\,\forall m\in [1,M]$

In equation 8, there is a missing comma after the first colon. It incorrectly reads ${{{\bf{O}}}}={{{\rm{sigmoid}}}}({{{{\bf{H}}}}}^{r(N)}[:{n}_{{{{\rm{emb}}}}}:])$ and it should read ${{{\bf{O}}}}={{{\rm{sigmoid}}}}({{{{\bf{H}}}}}^{{\prime} (N)}[:,{n}_{{{{\rm{emb}}}}}:])$

These errors have been corrected in the PDF and HTML versions of the Article.

