# Supplementary material for: Synthesize high-dimensional longitudinal electronic health records via hierarchical autoregressive language model
Source: Nat Commun. 2023 Aug 31;14:5305. doi: 10.1038/s41467-023-41093-0 (PMC10471716; doi:10.1038/s41467-023-41093-0)
Supplement: Supplementary file 1 — Supplementary Information [file 41467_2023_41093_MOESM1_ESM.pdf]

Supplementary Information for "Synthesize High-dimensional Longitudinal Electronic Health Records via Hierarchical Autoregressive Language Model"

Brandon Theodorou[1,2], Cao Xiao[2], Jimeng Sun[1,2*]
University of Illinois at Urbana-Champaign, 201 North Goodwin Avenue, Urbana, IL 61801[1]
Medisyn Inc., Las Vegas, NV[2]
* To whom correspondence should be addressed: jimeng@illinois.edu

We aim to make our main paper self-contained, comprehensive, and easy to understand by providing a detailed account of our task, the proposed HALO method, and the experimental results. In order to ensure greater completeness and clarity, we have included additional model details, dataset information, and results in this section.

1 SUPPLEMENTARY NOTE 1

One of the main contributions of HALO is its ability to provide strong performance in the high-dimensional setting found with real-world EHRs. In our main paper, we provide a comparison with the relatively low dimensionalities of some popular previous works in this domain. Here we provide a comprehensive comparison to all previous works we could find a dimensionality count for in Supplementary Table 1.

Method	Dimensionality
CONAN [1]	128*
CorGAN [2]	1,071*
EHR-M-GAN [3]	98
EMR-WGAN [4]	944*
EVA [5]	–^
HGAN [6]	926*
MedGan [7]	615*
MedWGAN [8]	1,651*
SynTEG [9]	1,276
HALO	9,882

Supplementary Table 1: Dimensionalities for previous ML approaches for generating synthetic EHR Data. * signifies a non-longitudinal output (producing either a patient embedding or a single aggregated vector instead of a series of visits) while ^signifies the special case of one-hot vector output that can only generate a limited number of common code combinations per visit predefined based on patterns from the training EHR data. No past approaches have ever produced synthetic health record data matching the high-dimensionality (on the order of 10,000+ medical codes).

2 SUPPLEMENTARY METHODS

2.1 Notation

We provide a table of notations for reference in Supplementary Table 2

Supplementary Table 2: Table of Notations

Notation	Description
$\mathcal{R}$	A patient's EHR medical record
$\mathcal{V}^{(t)}$	The t-th visit in $\mathcal{R}$
$m_i^{(t)}$	The i-th medical code in $\mathcal{V}^{(t)}$
$l_j^{(t)}$	The j-th lab value in $\mathcal{V}^{(t)}$
$g^{(t)}$	The gap between the $t-1$ and t-th visits
$\mathcal{S}$	A patient's static demographic information
$\mathcal{D}$	A patient's chronic disease information
$\mathcal{L}$	The set of all labs
$T \in \mathbb{N}$	The number of visits in $\mathcal{R}$
C	The set of all medical codes
$\mathbf{R} \in \mathbb{R}^{(T+3)\times\|C\|}$	The matrix representation of $\mathcal{R}$, $\mathcal{S}$, and $\mathcal{D}$
$\mathbf{v}_t \in \mathbb{R}^{\|C\|}$	The vector representation of the t-th visit in $\mathbf{R}$
$c_t^i \in \{0,1\}$	The binary presence of the i-th code in C in $\mathbf{v}_t$

2.2 Transformer Decoder Block

The visit-level module described in the main paper makes use of a stack of Transformer Decoder blocks from the original Transformer paper [10]. We provide additional details on those blocks here. Each block is defined mathematically by

$$\begin{aligned}
\mathbf{H}_1^{(m)} &= \mathbf{H}^{(m-1)} + \text{Masked Multi-Head Self-Attention}(\mathbf{H}^{(m-1)}) \\
\mathbf{H}_2^{(m)} &= \text{Layer Normalization}(\mathbf{H}_1^{(m)}) \\
\mathbf{H}_3^{(m)} &= (\mathbf{H}_2^{(m)} + (\max(0, \mathbf{H}_2^{(m)}\mathbf{W}^{(m)} + \mathbf{b}^{(m)})\mathbf{V}^{(m)} + \mathbf{c}^{(m)})) \\
\mathbf{H}^{(m)} &= \text{Layer Normalization}(\mathbf{H}_3^{(m)})
\end{aligned} \tag{1}$$

where Masked Multi-Head Self-Attention (MMSA) is then defined by

$$\begin{aligned}
\text{MMSA}(\mathbf{V}) &= \text{Concat}(head_1, \cdots, head_h)\mathbf{W}^O \\
head_i &= \text{Masked Attention}(\mathbf{V}\mathbf{W}_i^Q, \mathbf{V}\mathbf{W}_i^K, \mathbf{V}\mathbf{W}_i^V) \\
\text{Masked Attention}(\mathbf{Q}, \mathbf{K}, \mathbf{V}) &= \text{softmax}\left(\frac{\mathbf{Q}\mathbf{K}^T}{\sqrt{d_k}} + \mathbf{M}\right)\mathbf{V}
\end{aligned} \tag{2}$$

with $\mathbf{M}$ in the final line being a triangular matrix of $-\infty$ values ensuring that the softmax calculation only allows elements in the sequence to attend to themselves and elements before them.

Layer Normalization is then a regularization technique that ensures that all neurons or variables in the corresponding intermediate layer have the same distribution across all features in a given input, which has been shown to produce smoother gradients and more effective training.

2.3 Padding, Masking, and Truncating

Each patient may have a different number of visits in their medical record. We first limit the records to a maximum of 96 visits as an arbitrary number which requires a small percentage of truncations and results in sequences that are just under 100 visits after adding the first visit, label visit, and end visit. From there, to support batch input to a variety of models, we need to maintain a uniform size. So, we pad each record $\mathbf{R}$ of real length n to a constant number of columns, N, by appending the requisite number of empty vectors to $\mathbf{R}$. However, these padded visits as well as the initial start token visit do not contain any actual patient information and so should not be learned by any models. So, when calculating losses during training, both these ground truth visits and any outputs in these visits are zeroed out in order to ensure that they match and no loss arises from these meaningless visits.

2.4 Chronic Diseases Labels

Here we provide the specific list of chronic diseases we use to label patients in either dataset and also provide counts of how many patients possess each label.

The diseases for the outpatient dataset are:
- Alzheimer
- Kidney Disease
- Heart Failure
- Cancer
- Depression
- Arthritis
- COPD
- Stroke
- Heart Disease
- Diabetes
- Osteoporosis

The diseases for the inpatient dataset are:
- Renal Failure
- Cerebrovascular Disease
- Myocardial Infarction
- Cardiac Dysrhythmias
- Kidney Disease
- COPD
- Surgical Complications
- Conduction Disorders
- Congestive Heart Failure
- Heart Disease
- Diabetes With Complications
- Diabetes Without Complications
- Lipid Metabolism Disorders
- Essential Hypertension
- Fluid and Electrolyte Disorders
- Gastrointestinal Hemorrhage
- Hypertension with Complications
- Other Liver Diseases
- Other Lower Respiratory Diseases
- Other Upper Respiratory Diseases
- Pleurisy
- Pneumonia
- Respiratory Failure
- Septicemia
- Shock

The patient counts for the outpatient and inpatient datasets can be found in Supplementary Table 3 and 4 respectively.

2.5 Lab Values

In our evaluation of HALO's ability to handle and produce continuous values, we explored including lab measurements from an expanded inpatient dataset. Here we provide the specific list of those labs:
- Capillary Refill Rate
- Glascow Coma Scale Eye Opening
- Glascow Coma Scale Motor Response
- Glascow Coma Scale Verbal Response
- Glascow Coma Scale Total
- Diastolic Blood Pressure
- Systolic Blood Pressure
- Mean Blood Pressure
- Fraction Inspired Oxygen
- Glucose Percentage
- Heart Rate
- Height
- Weight
- Oxygen Saturation
- Respiratory Rate
- Temperature
- pH

3 SUPPLEMENTARY DISCUSSION

3.1 Code Orderings

As mentioned in our main paper, we explore different orderings of code variables within a visit but find limited effect, so we settle on a random ordering in our experiments. However, we provide the full test set modeling (F1 Score and Perplexity) results here for four different orderings on the inpatient dataset. Note that these orderings do not affect any baselines as they all model each code independently rather than with any intra-visit modeling which could be affected by the order, so we only include results from HALO here. We display results using our original ordering from the main paper, a second random ordering, alphanumeric ordering, and orderings low-to-high and high-to-low with respect to code prevalence in Supplementary Table 5. There we see relatively little impact from any of the different orderings considered, with the random ordering and pseudo-random alphanumeric orderings performing the best, validating our decision to proceed with a random order in our experiments.

3.2 Code Probabilities

While we capture the unigram, sequential visit bigram, and co-occurrence bigram probabilities normalized at both the visit and record levels, we presented only those normalized at the record level (the probability of a given patient having that code or pair of codes) in our main text beyond a table of just the R^2 values for the

	Alzheimer	Kidney Disease	Heart Failure	Cancer	Depression	Arthritis	COPD	Stroke	Heart Disease	Diabetes	Osteoporosis
Patients	6,325	30,357	50,242	83,117	117,839	1,997	105,547	58,305	23,926	155,463	23,325

Supplementary Table 3: Patient counts by disease label for the outpatient EHR dataset

	Renal Failure	Cerebrovascular Disease	Myocardial Infarction	Cardiac Dysrhythmias	Kidney Disease	COPD	Surgical Complications	Conduction Disorders	Congestive Heart Failure	Heart Disease	Diabetes With Complications	Diabetes Without Complications	Lipid Metabolism Disorders
Patients	9,549	3,679	5,032	13,702	5,039	5,090	10,853	2,976	10,432	13,114	3,413	8,061	12,248

	Essential Hypertension	Fluid and Electrolyte Disorders	Gastrointestinal Hemorrhage	Hypertension with Complications	Other Liver Diseases	Other Lower Respiratory Diseases	Other Upper Respiratory Diseases	Pleurisy	Pneumonia	Respiratory Failure	Septicemia	Shock
Patients	17,921	12,730	3,431	4,889	4,156	2,732	1,852	4,652	6,558	9,883	6,925	4,002

Supplementary Table 4: Patient counts by disease label for the inpatient EHR dataset

Ordering	F1 Score	PP Per Code
Main Paper (Random)	0.414	24.664
Another Random	0.419	23.120
Alphanumeric	0.417	23.680
High-to-Low Prevalence	0.404	24.680
Low-to-High Prevalance	0.400	27.243

Supplementary Table 5: The test set modeling results in terms of F1 Score and Perplexity achieved by HALO in the context of our original and four new code variable orderings. Here we see very limited impact from the different orderings, with the random ordering and pseudo-random alphanumeric orderings performing the best, validating the decision to proceed with a random ordering within the main paper.

visit-level due to the large size of the figure and the redundancy of further similar results. However, we feel that the visit level results are not only important but also offer insight into one of the key failure modes of the synthetic baselines and so provide them here. We show the visit level code probabilities in Supplementary Figure 1. There we see that at the visit level, each model is able to show strong correlation in the unigram evaluations, and the generally weaker performing baselines of SynTEG, EVA, and LSTM actually outperform the large language model baselines. SynTEG even improves upon HALO's R^2 correlation by 0.001 with those two offering state of the art results in the evaluation. However, those three baselines then struggle and the gap between HALO and all baselines widens in the sequential visit bigram probabilities. There SynTEG, EVA, and LSTM show basically no correlation while the GPT and HALO – Coarse baselines offer significantly less correlation than HALO. This signifies a significant lack of temporal coherence in each of the baselines compared to HALO. This incoherence is then a major failure mode and explains the highly sloped lines in many of the record-level code plots. Instead of repeating codes and maintaining consistency from one visit to the next over the course of a patient's medical history, many of those baselines produce individual visits which are reasonable but which are generated largely independently of another. This then causes the distinct number of codes and pairs of codes to be much larger than in real, temporally coherent patient data (and also explains why those steeply sloped plots are problematic and not easily fixed despite seemingly signaling correlation). The co-occurrence bigram results then largely mirror those in the record level plots albeit with slightly less correlation across the board due to the greater difficulty of the task. Again, HALO easily outpaces each of the baselines with SynTEG and GPT's inter-visit modeling placing them second and third with all others lagging significantly behind. So, in looking at the visit level code probabilities we are able to not only reconfirm HALO's state of the art performance but also gain insights into one of the major failure modes of the synthetic data baselines.

3.3 Low-Dimensional Setting

To offer a more ready-made comparison to the settings that past works were proposed within and to demonstrate how baseline performance degrades upon moving to the high-dimensional setting that we examine, we repeat some of our experiments on the outpatient EHR dataset converted to a lower dimensional setting. Specifically, we aggregate codes into code phenotypes and remove any phenotypes which show up in our dataset less than 1000 times in the same way as [9] did. This results in a low-dimensional version of our outpatient EHR dataset containing 1,349 distinct codes. We then train a new set of models and generate synthetic datasets for each of our compared methods of the same size as the training dataset. We extract a set of statistics and specifically examine a new set of per visit unigram, per visit bigram, and per visit sequential visit bigram code probabilities for each new low-dimensional synthetic dataset.

We present the plots of these probabilities compared to those in the low-dimensional training dataset in Supplementary Figure 2, and we compare the corresponding R^2 scores to those from the high dimensional setting in our main paper. We see that every model performs better and shows a stronger correlation for each probability than in the high-dimensional setting. The only exception to this universal improvement is SynTEG which performs slightly worse in the unigram and same-visit bigram code probabilities but with an extra boost in the more complex temporal patterns that it was specifically deficient in before as seen in its improved sequential visit bigram probabilities, chronic disease label probabilities, and

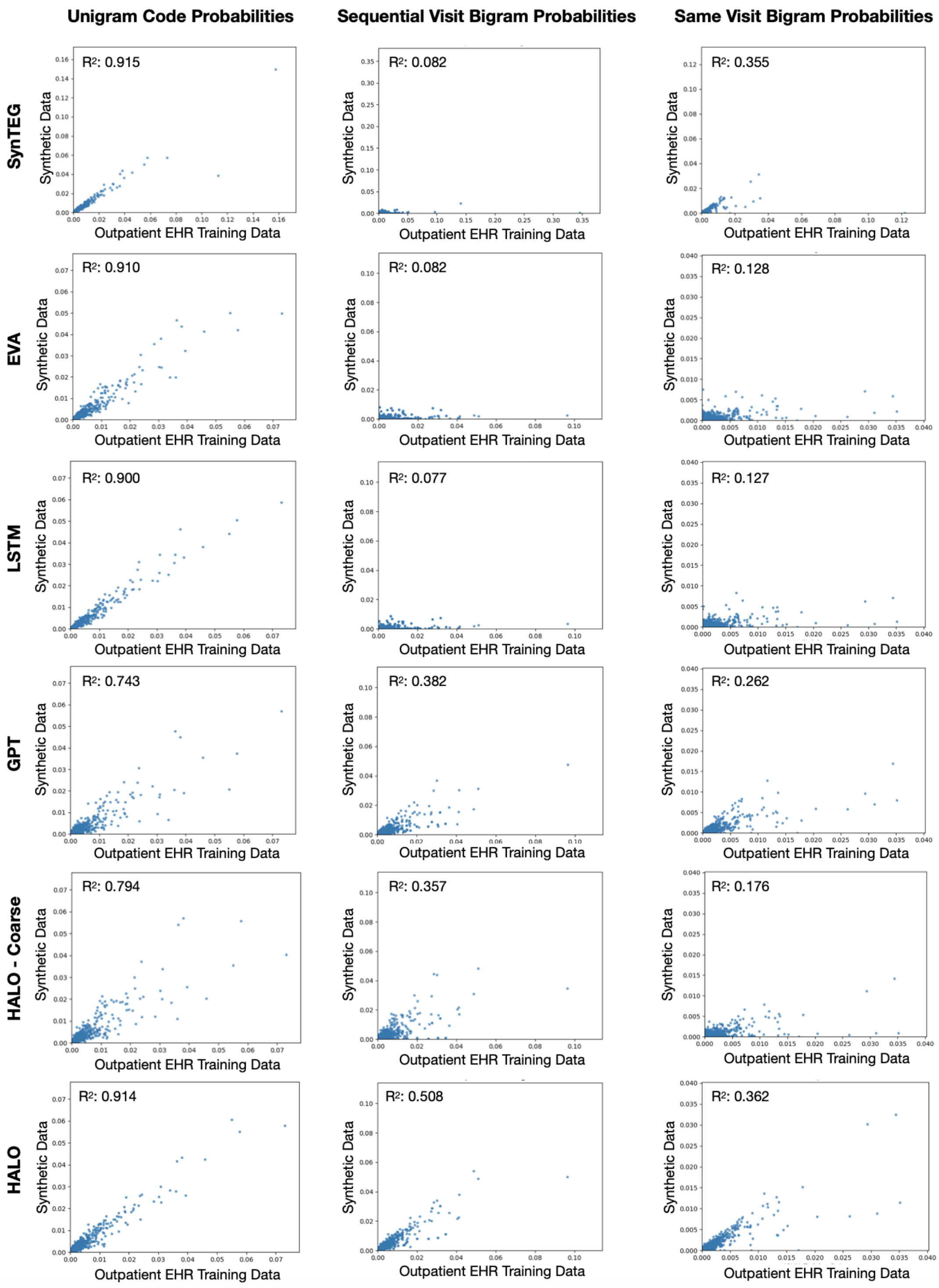

Supplementary Figure 1: Per Visit Unigram, Bigram, and Sequential Visit Bigram code probabilities for each synthetic dataset consisting of roughly 10,000, 1,500,000, and 5,000,000 points for each of the three types of plots respectively. These are analogous to the plots in the main paper but normalized at the probability of each code or pair of codes for individual visits rather than individual patients. Each model is able to show strong correlation in the unigram evaluations, and the generally weaker performing models of SynTEG, EVA, and LSTM perform very well. However, all of the baselines perform significantly worse in the sequential visit bigram probabilities, signaling their weaker temporal coherence which acts as a major failure mode for them. HALO is able to largely avoid that problem and maintain not only its temporal coherence but achieve state of the art results in all settings.

record shapes, making for much better performance overall. In this low-dimensional setting, some of the weaker baselines are even able to approach or slightly surpass HALO with respect to their per visit unigram code probabilities, demonstrating the strong performance that is showcased in their respective papers. However, those same baselines are then the ones that have the sharpest drop-off in performance when translating to the high-dimensional setting while HALO, HALO – Coarse, and GPT are able to maintain stronger performance there. Furthermore, those weaker baselines, HALO – Coarse, and to a lesser degree GPT all perform significantly worse in both of the more complex and difficult bigram probabilities even at this lower dimensionality. HALO is able to largely avoid that problem and maintain its performance there. So, HALO achieves very strong unigram code probabilities and state of the art results in the two bigram probabilities by a wide margin in this low-dimensional setting as well.

3.4 Comparison to ChatGPT

While we focus on comparing HALO to other leading baselines designed specifically for synthetic EHR generation, we feel that it is also worthwhile to compare its effectiveness to general purpose large language models such as OpenAI's ChatGPT. Such models have gained a lot of recent renown for offering state of the art performance in a wide variety of tasks. So, we explored ChatGPT's ability to generate patient records in a variety of formats and were impressed by its plausibility and ability to produce a variety of types of records (both in terms of conditional generation and output format). However, it is notable that despite this impressive ability, the generation process is designed to generate realistic responses overall rather than sample from the underlying distribution of patient records (let alone the distribution of records within a specific training dataset). This difference is then notable in preventing downstream usage for data analysis or machine learning tasks. To demonstrate the divide, we generate 1,000 patient records by feeding ChatGPT the prompt "Generate a realistic patient record as a sequence of hospital visits with a set of ICD-9 codes at each visit in the form [[Code1, Code2, ...], [Code3, ...], ...]". We then process and compare this synthetic dataset's statistics to those of our inpatient dataset. We show Per Visit code probabilities for both our HALO and ChatGPT in Supplementary Figure 3. While the fact that ChatGPT offers any correlation is an impressive feat, it is nonetheless clear that such language models can not be reasonably compared to synthetic EHR generation models such as HALO for the types of downstream use cases that are desired.

3.5 Record Shapes

We presented aggregate statistics regarding mean number of visits per record and codes per visit for both the real and synthetic datasets in our main paper. We now present the more detailed corresponding probability density plots for each statistic in Supplementary Figure 4. There we see that HALO and HALO- Coarse outperform the other baselines to accurately mimic the training dataset's shape not just on average but throughout the distribution.

We also present the chronic disease label probabilities for the outpatient dataset in Supplementary Figure 5. There EVA and HALO perform very well, effectively capturing the patterns of those codes found in the second, label visit.

3.6 Synthetic Training

Finally, we presented aggregated results across the chronic disease labels for both the outpatient and inpatient EHR dataset in our main paper as they are more concise and easier to understand. However, for the sake of completeness we also provide accuracy by individual label for models trained on each of our compared synthetic datasets and the real training dataset in Supplementary Table 6 for our outpatient EHR dataset and Supplementary Table 7 for our inpatient EHR dataset.

We then also provide additional aggregated results on the outpatient data demonstrating the effect of using synthetic data as an augmentation technique to supplement real data. Specifically, we add additional models for each chronic disease classification task trained on the same real data but augmented with each synthetic datasets in turn as well. We show those aggregated results of mean test set classification performance across the 11 label-based tasks in Supplementary Table 8. These results mirror those from the original setting of replacing real training data with synthetic data, with HALO performing the best. However, it here offers the most gain over real-only training datasets rather than the least dropoff. Thus, we show that HALO's synthetic data is able to be effectively used as an augmentation technique to produce better results than can be achieved with real data alone.

3.7 Additional Privacy Evaluations

In our main paper we use a pair of membership inference attacks to evaluate the privacy preservation of HALO and our other compared methods. Here we provide two other attacks from literature and show that HALO thwarts them as well.

Attribute Inference Attack: The first of the two additional evaluations is the ability to thwart a typical attribute inference attack. This attack determines whether the synthetic dataset leaks specific and sensitive patient attributes based on correlations from demographic and other more common, less sensitive attributes of the patient. Consequently, it tests whether the synthetic dataset can be used to learn individual attributes of real patient data.

To demonstrate that HALO is not susceptible to such an attack, we show that it thwarts the nearest neighbor-based attribute inference attack. In this attack, we use subsets of the synthetic dataset and the original training dataset, randomly sampled to match the size of the original test dataset. We define demographic information, chronic disease labels, and the binary presence of the 500 most common medical codes (determined by the training dataset) as the conditional attributes. The sensitive attributes to be identified are the binary presence of the remaining uncommon medical codes.

To conduct the attack, we find the closest patient in the synthetic dataset for each patient in the training set based on having the most shared conditional attributes. We then predict each of the uncommon attributes to be the same as that closest synthetic patient. Those predicted attributes are compared with the ground truth sensitive patient attributes and graded using F1 Score. We then repeat this attack with real patients from the test dataset in place of

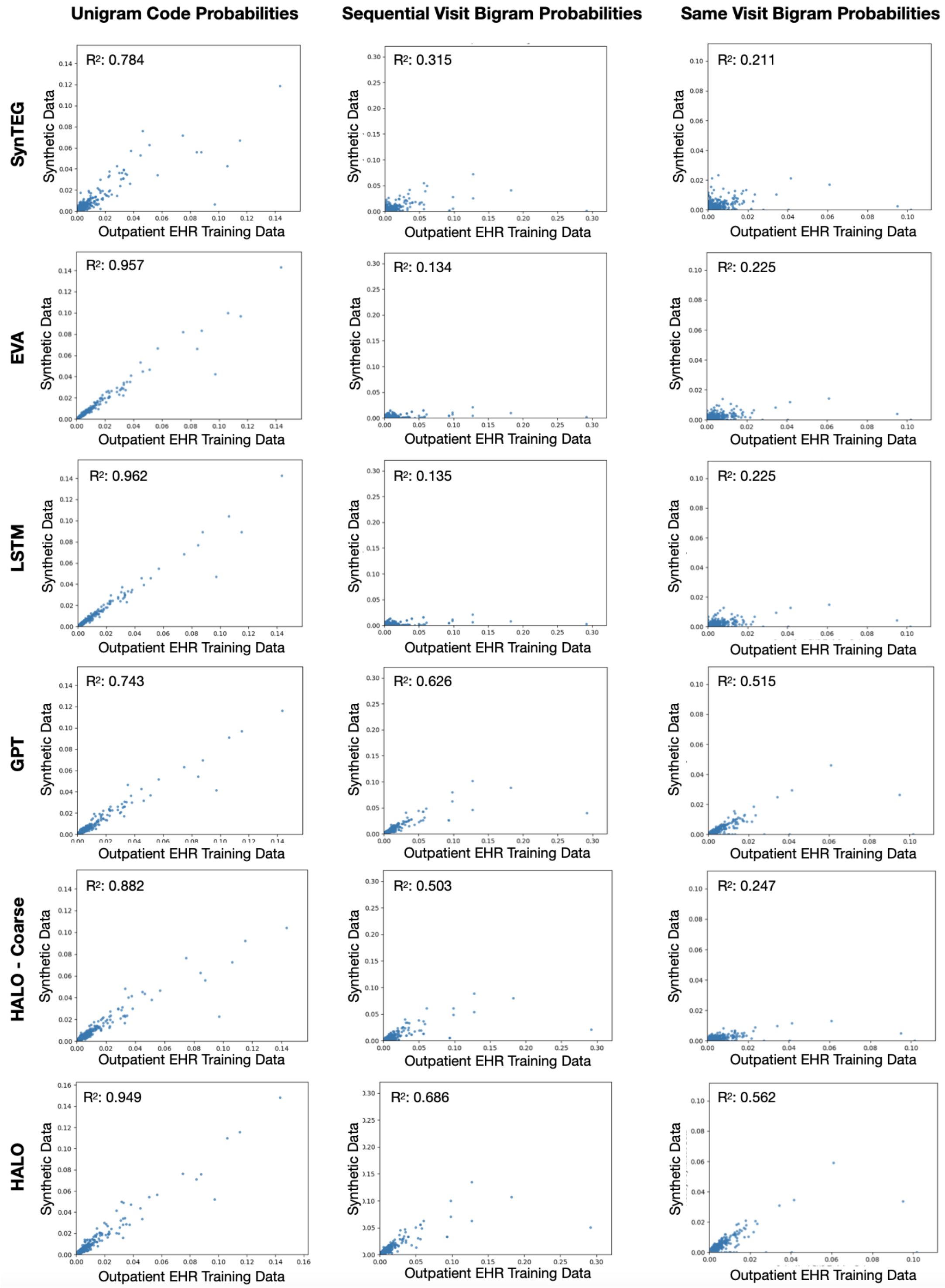

Supplementary Figure 2: Per Visit Unigram, Bigram, and Sequential Visit Bigram code probabilities for each synthetic dataset in the lower-dimensional setting consisting of just around 1,300 code phenotypes after code aggregation and rare code removal. These are the same plots as Supplementary Figure 1 but in a low-dimensional setting to offer a comparison with the original setting of past works. Each model performs better and shows stronger correlation for each probability than in the high-dimensional setting. Some of the weaker baselines are even able to approach or slightly surpass HALO with respect to their unigram code probabilities. However, all of the baselines perform significantly worse in both bigram probabilities even at this lower dimensionality. HALO is able to largely avoid that problem and maintain its performance for those more complex probabilities. So, HALO achieves very strong unigram code probabilities and state of the art results in the two bigram probabilities in this low-dimensional setting as well.

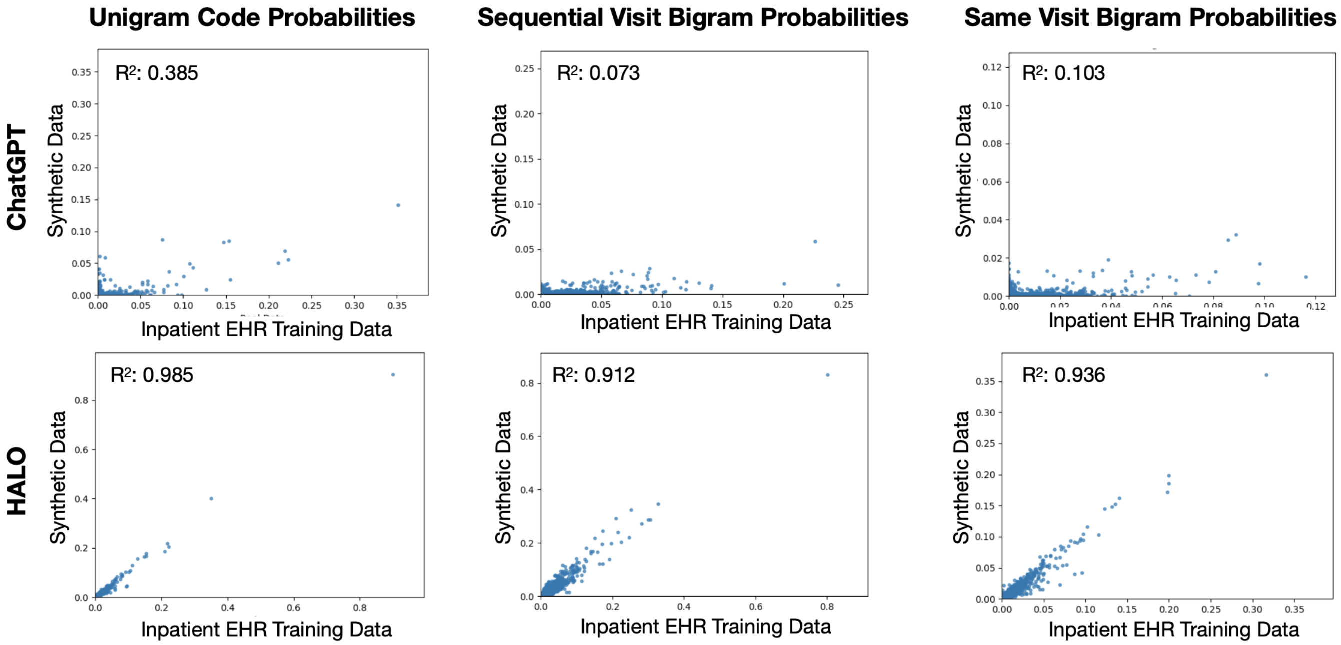

Supplementary Figure 3: Per Visit Unigram, Bigram, and Sequential Visit Bigram code probabilities for HALO and ChatGPT's synthetic inpatient datasets. While ChatGPT's responses and data appear reasonable, they are not data-driven in terms of sampling from the underlying distribution of patient records and offer little correlation. HALO is alternatively able to closely mirror the true distribution and statistics.

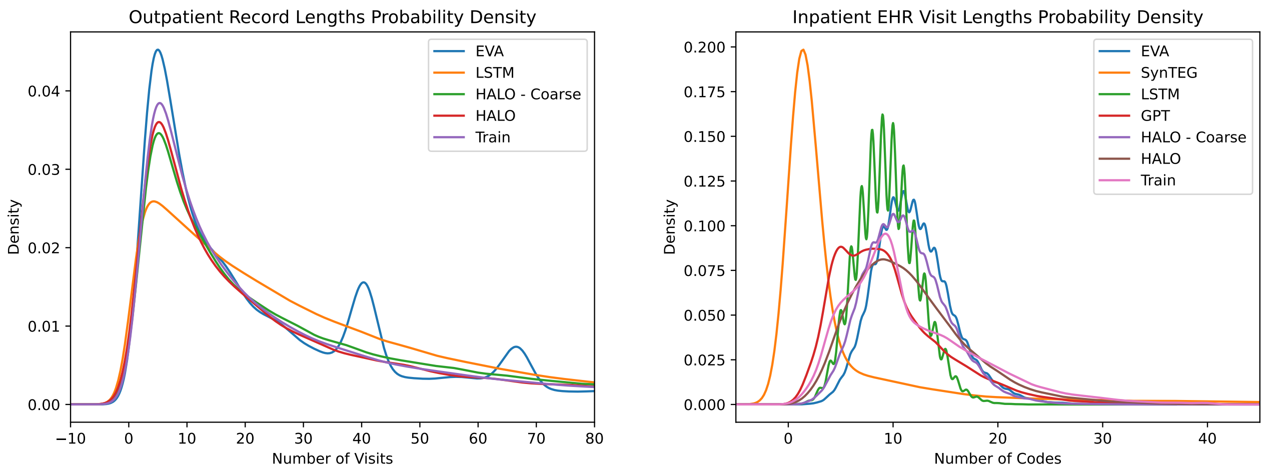

Supplementary Figure 4: Probability densities for the number of visits per patient record for the outpatient EHR dataset and corresponding synthetic datasets and the number of codes per visit for the inpatient EHR dataset and corresponding synthetic datasets respectively. We see that the language model architectures such as HALO and HALO − Coarse outperform the other baselines to accurately mimic the training data distribution.

the synthetic dataset and use the results as a baseline for acceptable attribute inference.

We show the results of the classifications from the nearest neighbor attacks in Supplementary Table 9. There we see that not only are the prediction F1 Scores incredibly low on both datasets (4.7% for the outpatient dataset and 3.3% for the inpatient dataset), they are crucially lower than the baseline attack from the test set. This attack, labeled Real Data Attack in the table, sets the threshold for the amount of information revealed by the patterns of real data. So, staying below that level means incurring only an acceptable amount of attack success. So, we see that the synthetic dataset does not reveal any meaningful insight into the attributes of real patient

	Alzheimer	Kidney Disease	Heart Failure	Cancer	Depression	Arthritis	COPD	Stroke	Heart Disease	Diabetes	Osteoporosis
EVA	0.518	0.533	0.5	0.549	0.449	0.5	0.559	0.5	0.526	0.5	0.5
SynTEG	0.494	0.516	0.580	0.498	0.438	0.500	0.572	0.460	0.512	0.500	0.502
LSTM	0.574	0.492	0.499	0.508	0.445	0.475	0.5	0.499	0.499	0.5	0.500
GPT	0.908	0.903	0.904	0.857	0.829	0.844	0.861	0.846	0.911	0.951	0.851
HALO – Coarse	0.915	0.905	0.909	0.891	**0.854**	**0.930**	0.937	0.885	0.914	0.951	0.888
HALO	**0.924**	**0.909**	**0.912**	**0.908**	0.852	0.926	**0.948**	**0.889**	**0.921**	**0.963**	**0.896**
Real	0.953	0.936	0.940	0.931	0.901	0.952	0.945	0.924	0.928	0.966	0.922

Supplementary Table 6: Full accuracy results by compared method and chronic disease label for the outpatient EHR dataset

	Renal Failure	Cerebrovascular Disease	Myocardial Infarction	Cardiac Dysrhythmias	Kidney Disease	COPD	Surgical Complications	Conduction Disorders	Congestive Heart Failure	Heart Disease	Diabetes With Complications	Diabetes Without Complications	Lipid Metabolism Disorders	Essential Hypertension	Fluid and Electrolyte Disorders	Gastrointestinal Hemorrhage	Hypertension with Complications	Other Liver Diseases	Other Lower Respiratory Diseases	Other Upper Respiratory Diseases	Pleurisy	Pneumonia	Respiratory Failure	Septicemia	Shock
EVA	0.52	0.50	0.59	0.49	0.58	0.58	0.52	0.60	0.55	0.49	0.50	0.63	0.52	0.46	0.50	0.52	0.50	0.56	0.58	0.41	0.50	0.50	0.51	0.59	0.58
SynTEG	0.56	0.61	0.51	0.46	0.65	0.49	0.51	0.63	0.54	0.50	0.49	0.56	0.73	0.50	0.52	0.57	0.58	0.50	0.54	0.52	0.44	0.60	0.55	0.44	0.50
LSTM	0.54	0.50	0.55	0.53	0.58	0.49	0.49	0.48	0.57	0.58	0.46	0.56	0.50	0.56	0.49	0.52	0.55	0.49	0.47	0.48	0.53	0.50	0.57	0.38	0.56
GPT	0.90	0.86	**0.91**	0.90	**0.93**	0.88	0.85	**0.83**	**0.95**	**0.93**	0.87	0.89	**0.93**	0.91	0.83	0.84	0.90	0.85	0.72	0.73	**0.86**	**0.87**	**0.86**	**0.92**	0.88
HALO – Coarse	**0.91**	0.86	0.81	0.90	0.87	**0.89**	0.84	0.82	**0.95**	0.90	0.87	**0.91**	0.86	**0.94**	**0.84**	**0.86**	0.89	0.86	0.69	0.73	0.85	0.86	0.81	0.91	0.83
HALO	0.90	**0.89**	0.90	**0.91**	0.91	0.88	**0.87**	0.82	0.92	**0.93**	**0.89**	**0.91**	0.92	0.93	**0.84**	0.84	**0.93**	**0.87**	**0.74**	**0.80**	0.85	0.85	0.85	0.90	**0.89**
Real	0.95	0.95	0.96	0.96	0.95	0.96	0.91	0.93	0.96	0.95	0.94	0.96	0.96	0.97	0.89	0.94	0.96	0.90	0.81	0.89	0.90	0.94	0.89	0.97	0.95

Supplementary Table 7: Full accuracy results by compared method and chronic disease label for the inpatient EHR dataset

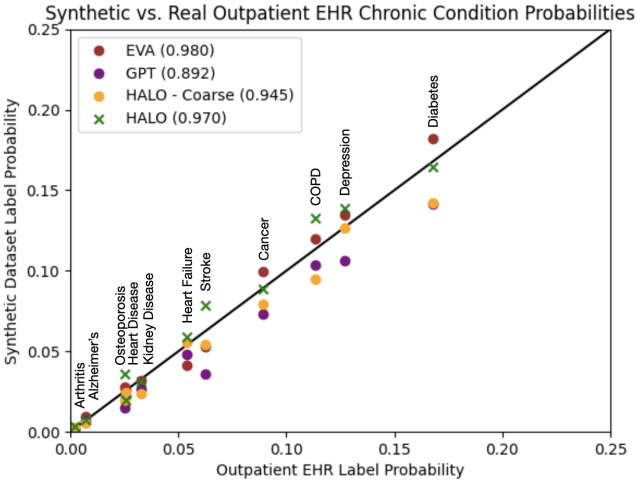

Supplementary Figure 5: We plotted the probabilities of each chronic disease label in the original outpatient EHR training dataset against their corresponding probabilities in each synthetic dataset. The R^2 value is shown in parentheses in the legend. The SynTEG and LSTM baselines both struggle with temporal consistency as manifested through their weak ability to create these chronic disease labels in the label visit, so they are omitted from the plot. In contrast, the EVA, HALO – Coarse, and HALO architectures all closely mirror the training data with HALO and EVA performing the best overall on average.

	Avg. Accuracy	Avg. F1 Score	Avg. AUROC
EVA	0.508 ± 0.02	0.283 ± 0.26	0.471 ± 0.08
SynTEG	0.507 ± 0.03	0.514 ± 0.20	0.506 ± 0.07
LSTM	0.506 ± 0.02	0.467 ± 0.28	0.495 ± 0.06
GPT	0.851 ± 0.03	0.854 ± 0.03	0.914 ± 0.03
HALO – Coarse	0.867 ± 0.03	0.863 ± 0.03	0.920 ± 0.03
HALO	0.879 ± 0.03	0.878 ± 0.03	0.938 ± 0.02
Real Data	0.891 ± 0.03	0.895 ± 0.03	0.943 ± 0.02
EVA + Real	0.844 ± 0.03	0.852 ± 0.03	0.921 ± 0.02
SynTEG + Real	0.846 ± 0.03	0.850 ± 0.02	0.915 ± 0.02
LSTM + Real	0.853 ± 0.02	0.857 ± 0.03	0.923 ± 0.02
GPT + Real	0.904 ± 0.02	0.906 ± 0.02	0.953 ± 0.01
HALO – Coarse + Real	0.910 ± 0.02	0.910 ± 0.02	0.958 ± 0.01
HALO + Real	**0.912 ± 0.02**	**0.912 ± 0.02**	**0.959 ± 0.02**

Supplementary Table 8: Chronic disease classification models trained on different types of training data in the outpatient setting - real data, synthetic data generated by different methods, and real data augmented by synthetic data. Values are mean and standard error over 11 tasks. Bold values denote the best results. GPT, HALO – Coarse, and HALO's synthetic data perform better than the other methods, and are comparable to using real data as training data. Augmenting real data with HALO's synthetic data leads to better performance than just using real data. HALO has the best results, with little drop-off in performance compared to real data and the largest gain when used to augment the training set.

data. We then see that each of the baseline synthetic datasets pass the test as well by having lower F1 Scores than the real data attack.

	Outpatient EHR F1 Score	Inpatient EHR F1 Score
Synthetic Data Attack	0.0397	0.0335
Real Data Attack	0.0503	0.0473
EVA	0.0108	0.0078
SynTEG	0.0162	0.0094
LSTM	0.0119	0.0068
GPT	0.0447	0.0324
HALO − Coarse	0.0330	0.0202

Supplementary Table 9: The results of the nearest neighbor attribute inference attack. The results showed that the F1 Score on both the inpatient and output datasets was below 0.05, and crucially lower than the baseline attacks using real data from the test set. This baseline attack sets the threshold for the amount of information revealed by the patterns of real data and so staying below it means incurring only an acceptable amount of attack success. This suggests that the synthetic dataset does not reveal any significant insights into the attributes of real patient data, and that HALO is effective in preventing an attacker from inferring sensitive information. We then see that each of the baseline synthetic datasets pass the test as well by having lower F1 Scores than the real data attack. GPT and HALO − Coarse allow similar F1 Scores to HALO while all of the rest have much lower scores, likely because they do not capture the real patterns as effectively. Source data are provided as a Source Data file.

GPT and HALO − Coarse allow similar F1 Scores to HALO while all of the rest have much lower scores, likely because they do not capture the real patterns as effectively.

Nearest Neighbor Adversarial Accuracy Risk: The final evaluation, proposed in [11], measures the degree to which a model overfits to its training dataset by looking at the relative likelihood of a patient's nearest neighbor being in the same or different datasets. As such, passing this test ensures that a generative model is generating wholly new synthetic patients rather than copying or performing simple augmentation on real training patients.

The evaluation is performed by calculating the metric Nearest Neighbor Adversarial Accuracy (NNAA). Let S_T, S_S, and S_E be random subsets of n records (we use n = 5,000 records in our experiment) from the training, synthetic, and evaluation datasets respectively. NNAA risk is then the difference

$$AA_{ES} - AA_{TS} \quad (3)$$

where

$$AA_{ES} = \frac{1}{2}\left(\frac{1}{n}\sum_{i=1}^{n} 1\left(d_{ES}(i) > d_{EE}(i)\right) + \frac{1}{n}\sum_{i=1}^{n} 1\left(d_{SE}(i) > d_{SS}(i)\right)\right)$$
$$AA_{TS} = \frac{1}{2}\left(\frac{1}{n}\sum_{i=1}^{n} 1\left(d_{TS}(i) > d_{TT}(i)\right) + \frac{1}{n}\sum_{i=1}^{n} 1\left(d_{ST}(i) > d_{SS}(i)\right)\right) \quad (4)$$

where the E subscript throughout refers to the evaluation (test) dataset, S refers to the synthetic dataset, and T refers to the training dataset. $1(\cdot)$ is then the indicator function and $d_{ES}(i)$ is the distance from the i-th record in the evaluation dataset to its closest record (as determined by hamming distance in accordance with [12]) in the synthetic dataset. Each of E and S in $d_{ES}(i)$ can also be replaced interchangeably with any of E, S, and T, where the calculation just omits the record in question if the two datasets are the same. So, each $\frac{1}{n}\sum_{i=1}^{n} 1(d_{AB}(i) > d_{AA}(i))$ component is the probability of a record in dataset A being closer to another record in its own dataset than any record in dataset B. If they are randomly drawn from the same or similar distributions, we would expect that probability to be $\frac{1}{2}$, but it could be much lower if one of the datasets were copying from the other. We baseline this likelihood of the synthetic dataset copying from both its training and testing datasets, comparing the two to produce our overall risk.

[11] set 0.03 as the threshold for an acceptable NNAA risk. We show in Supplementary Table 10 that the NNAA values for both our inpatient and outpatient datasets are easily below that mark. Furthermore, we show that as more data is added as with the outpatient EHR dataset, the risk decreases to an even smaller value. So, we show that our HALO method is not overfitting to or copying from its training dataset and instead is producing wholly new synthetic records. We repeat the evaluation with each of the baseline synthetic datasets and show that they pass as well.

So, HALO succeeds in passing both of our additional privacy evaluations, further reinforcing that its strong performance does not come at the expense of the privacy of the underlying patient records.

Method	Outpatient NNAA	Inpatient NNAA
HALO	0.0104	0.0211
EVA	0.0040	0.0018
SynTEG	-0.0002	-0.0080
LSTM	0.0178	0.0082
GPT	0.0045	0.0221
HALO − Coarse	0.0047	0.0301

Supplementary Table 10: The results of the nearest neighbor adversarial accuracy risk evaluation. These values are calculated through the likelihood of data in the synthetic dataset being overly similar to records in the training set, normalized by their baseline likelihood of being close to unseen test set data. The metric was proposed in [11] where they set 0.03 as the acceptable risk threshold, a value that both the inpatient and outpatient synthetic datasets are well below. HALO and other baselines all achieve much lower NNAA risk.

SUPPLEMENTARY REFERENCES

[1] Cui, L. *et al.* Conan: Complementary pattern augmentation for rare disease detection. In *Proceedings of the AAAI Conference on Artificial Intelligence*, vol. 34, 614–621 (2020).

[2] Torfi, A. & Fox, E. A. Corgan: Correlation-capturing convolutional generative adversarial networks for generating synthetic healthcare records. In *The Thirty-Third International Flairs Conference* (2020).

[3] Li, J., Cairns, B. J., Li, J. & Zhu, T. Generating synthetic mixed-type longitudinal electronic health records for artificial intelligent applications. *arXiv preprint arXiv:2112.12047* (2021).

[4] Zhang, Z., Yan, C., Mesa, D. A., Sun, J. & Malin, B. A. Ensuring electronic medical record simulation through better training, modeling, and evaluation. *Journal of the American Medical Informatics Association* **27**, 99–108 (2020).

[5] Biswal, S. *et al.* Eva: Generating longitudinal electronic health records using conditional variational autoencoders. In *Machine Learning for Healthcare Conference*, 260–282 (PMLR, 2021).

[6] Yan, C., Zhang, Z., Nyemba, S. & Malin, B. A. Generating electronic health records with multiple data types and constraints. In *AMIA Annual Symposium Proceedings*, vol. 2020, 1335 (American Medical Informatics Association, 2020).

[7] Choi, E. *et al.* Generating multi-label discrete patient records using generative adversarial networks. In *Machine learning for healthcare conference*, 286–305 (PMLR, 2017).

[8] Baowaly, M. K., Lin, C.-C., Liu, C.-L. & Chen, K.-T. Synthesizing electronic health records using improved generative adversarial networks. *Journal of the American Medical Informatics Association* **26**, 228–241 (2019).

[9] Zhang, Z., Yan, C., Lasko, T. A., Sun, J. & Malin, B. A. Synteg: a framework for temporal structured electronic health data simulation. *Journal of the American Medical Informatics Association* **28**, 596–604 (2021).

[10] Vaswani, A. *et al.* Attention is all you need. In *Advances in neural information processing systems*, 5998–6008 (2017).

[11] Yale, A. *et al.* Generation and evaluation of privacy preserving synthetic health data. *Neurocomputing* **416**, 244–255 (2020).

[12] Yan, C. *et al.* A multifaceted benchmarking of synthetic electronic health record generation models. *Nature Communications* **13**, 7609 (2022).
